# Detection and quantification of ergothioneine in human serum using surface enhanced Raman scattering (SERS)†

**DOI:** 10.1039/d4an01323a

**Published:** 2024-12-30

**Authors:** Stefano Fornasaro, Nigel Gotts, Gioia Venturotti, Marina Wright Muelas, Ivayla Roberts, Valter Sergo, Royston Goodacre, Alois Bonifacio

**Affiliations:** a Department of Chemical and Pharmaceutical Sciences, University of Trieste via L. Giorgieri 1 34127 Trieste Italy sfornasaro@units.it; b Centre for Metabolomics Research, Department of Biochemistry, Cell and Systems Biology, Institute of Systems, Molecular, and Integrative Biology, University of Liverpool Liverpool L69 7ZB UK; c Department of Engineering and Architecture, University of Trieste via A. Valerio 6 34127 Trieste Italy

## Abstract

Ergothioneine (ERG) is a natural sulfur-containing amino acid found in many organisms, including humans. It accumulates at high concentrations in red blood cells and is distributed to various organs, including the brain. ERG has numerous health benefits and antioxidant capabilities, and it has been linked to various human physiological processes, such as anti-inflammatory, neuroprotective, and anti-aging effects. Accurate, rapid, and cost-effective quantification of ERG levels in human biofluids is crucial for understanding its role in oxidative stress-related diseases. Surface-enhanced Raman scattering (SERS) is an effective approach for measuring compounds at concentrations similar to those at which ERG is present in serum. However, while SERS has been used to characterize or detect ERG, quantification has not yet been achieved due to the variability in the signal enhancement that can arise during sample preparation and analysis. This study introduces a highly efficient and reliable technique for quickly (20 min is typical per sample) measuring ERG levels in human serum using SERS. This employs an internal standard highly specific for ERG which resulted in limit of quantification values of 0.71 μM. To validate this approach, we analysed real human serum with unknown ERG levels as a blind test set and primary reference levels of ERG were produced using a targeted UHPLC-MS/MS reference method.

## Abstract 2

Ergothioneine (ERG) is a natural sulfur-containing amino acid linked to various human physiological processes, such as anti-inflammatory, neuroprotective, and anti-aging effects. ERG can be measured in human serum using SERS in < 20 min employing a highly specific internal standard.

## Graphical abstract

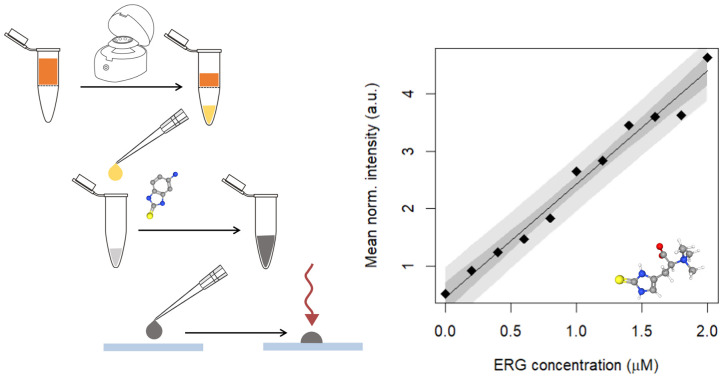

## Introduction

Ergothioneine (ERG) is a natural sulphur-containing amino acid present in many organisms, including humans, where it is derived primarily from the consumption of mushrooms.^1^ Through the action of a novel type-1 organic cation transporter, OCTN1, ERG is able to accumulate at relatively high concentrations in red blood cells (RBCs), and is distributed to several organs, including the brain, after consumption.^2^ Its clearance from the body is relatively slow.^3^ Due to its possible health benefits and outstanding antioxidant capabilities, it has garnered a lot of interest. Not only is ERG very resistant to heat and oxygen, but it is also very stable and easily soluble. Significant biological functions of this compound include protecting cells from photochemical damage, neutralizing harmful reactive oxygen and nitrogen species (ROS and RNS), and lowering the associated oxidative stress.^4^ Furthermore, ERG has been linked to numerous human physiological processes, such as anti-inflammatory, neuroprotective, and anti-aging effects.^5,6^ Consequently, ERG has gained popularity as a candidate for use as an ingredient in many different products and processes, such as pharmaceuticals, nutraceuticals, functional foods and drinks, cosmetics, and skincare.^2,7^ As research uncovers its full range of benefits and applications, the uses of ERG as an ingredient continue to expand. Given these diverse potential applications, there is a rising need for a precise and straightforward method to measure ERG in various complex matrices, including foods, cosmetics, and human biofluids. Accurate, rapid, and cost-effective quantification of ERG levels in biofluids is crucial for understanding its role in oxidative stress-related diseases, such as neurodegenerative disorders, cardiovascular diseases, and cancer,^1,7^ in large epidemiological studies. Existing analytical methods for ERG determination in biological samples include: (i) capillary electrophoresis;^8^ (ii) high-performance liquid chromatography (HPLC);^8,9^ (iii) liquid chromatography-inductively coupled plasma mass spectrometry (LC/ICPMS);^10^ (iv) liquid chromatography tandem mass spectrometry (LC-MS/MS) and isotope-dilution liquid chromatography tandem MS (ID-LC–MS/MS).^11^ However, these techniques all require complex and destructive sample preparation procedures, involve time-consuming separations with high-purity reagents. None of these assays has proved to be suitable for a convenient general use. Moreover, human serum contains 2 to 9-fold less ERG than erythrocytes, therefore, an improvement of the sensitivity in the detection system is required for its analysis in such matrix. One approach that is effectively used for measuring compounds at concentrations similar to those at which ERG is present in serum, employs surface-enhanced Raman scattering (SERS). SERS spectroscopy has emerged as a powerful analytical technique for the detection and quantification of various metabolites in the context of liquid biopsy.^12–14^ SERS exploits the phenomenon of enhanced Raman scattering signals when analytes are adsorbed (or very close to) onto nanostructured metal surfaces, such as gold or silver nanoparticles. The enhancement arises from the excitation of localized surface plasmon resonances on the metal nanoparticles, resulting in intensified Raman signals. SERS spectra of ERG have been recently reported from both aqueous solutions and RBCs lysates,^15^ and the presence of ERG bands has been reported in SERS spectra of many biological samples.^16^ So far, however, SERS has been used to characterize or detect ERG, but not to quantify it. One big challenge in accurate quantification using SERS is the variability that can arise during sample preparation and analysis.^17^ Factors such as variations in the SERS substrate, laser power, and experimental conditions can affect the Raman signal intensity, leading to potential inaccuracies in quantification. Including an internal standard (IS) of known concentration in the unknown sample and using its SERS spectral band intensity as a reference to the band intensity of the chemical of unknown concentration, is an effective way to overcome such variation errors. The variability in signal enhancement across different analytes makes it difficult to identify a single compound that can serve as an appropriate IS for all cases. Here we introduce a highly efficient and reliable technique for quickly measuring ERG levels in human serum. This method utilizes surface-enhanced Raman scattering and incorporates an internal standard that is specifically designed for ERG. The quantification of ERG was performed using the SERS method in a subset of 11 human serum samples from the Husermet study.^18^ This was done in parallel with a reference method (UHPLC-MS/MS) for benchmarking purposes. It is worth noting that the SERS quantification was carried out without any prior knowledge of the ERG levels (primary reference data) determined by UHPLC-MS/MS, following a “blind” approach. This is significant because the IS-SERS method discussed for ERG quantification in real samples is a pioneering study and has not been previously tested on human samples.

## Experimental section

### Materials and reagents

For SERS experiments, all chemicals (analytical grade) were purchased from Merck (Darmstadt, Germany) and used as received unless otherwise stated. l-(+)-Ergothioneine (ERG; purity >98.0%) was obtained from Merck. Ultrapure deionized (DI) water of 18.2 MΩ cm resistivity at 25 °C was used throughout the SERS experiments and it was obtained by a Millipore Milli-Q system (Merck, Germany). Phosphate buffered saline (PBS) solution (0.01 M, pH 7.4) was prepared by dissolving one PBS tablet in DI water (200 mL). For LC-MS, all solvents used were Fisher Optima LC/MS grade (Fisher Scientific, UK). The ERG (DRE-CA13202000 and TRC-E600000) used in method optimisation and standard addition method were obtained from LGC (UK); DRE-CA13202000 was used for method optimisation (due to a relatively short remaining shelf life), then TRC-E600000 was used for standard addition spike in the actual analysis. Both were of analytical grade and so this does not affect the LC-MS method.

### Stock solutions and internal standard preparation for SERS measurements

**Fig. 1:**
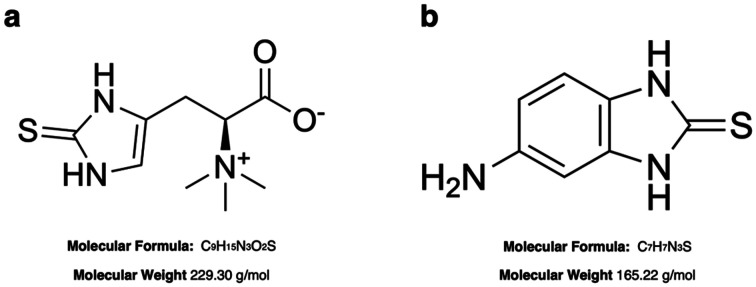
Molecular structures of ergothioneine (ERG, a) and 5-amino-2-mercaptobenzimidazole (5A2MBI, b).

A stock solution of ERG (10 mM) was prepared by dissolving 2.3 mg of ERG in 1 mL of PBS solution, and further desired concentrations were obtained by spiking ERG in commercial human serum or in PBS (pH 7.4) over the range from 0 to 10 μM. The aliquots were freshly prepared before each measurement. Stock solution of 5-amino-2-mercaptobenzimidazole (5A2MBI or IS) 10 mM was prepared dissolving 1.65 mg of powder in 1 mL of MeOH (HPLC grade). Fresh working solutions were prepared by diluting the stock solution with PBS to the required concentrations before use. The molecular structures of ERG and IS are presented in Fig. 1.

### Colloidal-based SERS substrates

Citrate-reduced silver nanoparticles (cAgNPs) were synthesized using the Lee–Meisel method.^19^ Briefly, 45 mg of AgNO_3_ were dissolved in 250 mL of DI water and heated to boiling and under magnetic stirring. Successively 5 mL of a 1% sodium citrate tribasic solution were added dropwise to the boiling solution. The solution was kept boiling and stirring for 1 h in conditions of complete darkness. The result is a greenish grey solution. The cAgNP were stored in dark at room temperature and were stable for months. All colloids have been characterized by UV-Visible absorption spectroscopy after each preparation, using UV-visible spectroscopy (Cary60, Agilent Technology). The extinction band maxima were between 405 and 410 nm, corresponding to an average particle size of 50 nm.

### SERS instrumentation

SERS spectra were collected at room temperature (22 ± 0.5 °C) with a portable i-Raman plus spectrometer (B&W Tek, Plainsboro, NJ, USA) equipped with a 785 nm laser and connected with a compact microscope mounting a 20× Olympus optics with a spot size of 108 μm (N.A. 0.40). The spectral acquisition was performed using the BWSpec™ version 4.03_23_c (B&WTek., Newark, DE, USA) software in the Raman shift range of 62–3202 cm^−1^. The BWSpec™ software allowed for the collection of a background signal (dark) before data acquisition and its spectral acquisition session by collecting a spectrum of paracetamol and silicon as standard references.

### Sample preparation and SERS assay

**Fig. 2:**
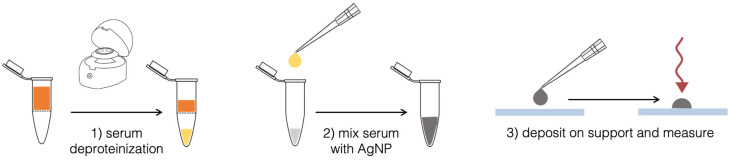
Schematic summary of the sample preparation protocol for SERS measurements.

Spiked serum samples were obtained by adding 2 μL of a variable concentration of ERG, obtained by diluting ERG stock solution with PBS, to 198 μL of human serum. For all samples, 2 μL of a 25 μM 5A2MBI (IS) solution, obtained by diluting IS stock solution with PBS, were added to 198 μL of serum spiked with ERG, to reach a final concentration of 0.25 μM. The SERS analysis protocol included a step of deproteinization using 3 kDa centrifugal filters (Fig. 2). This step improves the SERS signal from serum, as previously reported.^20,21^ The Vivaspin® filters (Sartorius, UK) were first rinsed with DI water and centrifuged two times at 11 337*g* for 15 min using a Minispin centrifuge (Eppendorf, Hamburg, Germany). Next, 200 μL of serum spiked with ERG at different concentrations (0.2–2 μM), and a blank sample, were added after this procedure and centrifuged for two cycles of 20 min at 8117*g*. 25 μL of the filtered samples were mixed with 25 μL of a colloidal dispersion of cAgNPs for further SERS analysis. After mixing, a change in the colour can be observed (*i.e.* a shift to a more blueish shading), indicating a partial nanoparticles aggregation due to the absorption of the analytes on the metal surface. The resulting 50 μL drop was rapidly deposited under the microscope objective on a glass microslide (25 × 75 mm), previously covered in aluminium foil and parafilm, that was fitted onto the portable microscope stage. The aluminium film was used to avoid spectral interference from fluorescence of the glass substrate. Moreover, the hydrophobic surface of parafilm avoided the spread of the liquid sample, inducing the formation of semi-spherical drop. Parafilm gives a Raman signal, which was however not detected with the confocal optics used, if the measurement is taken by focusing the laser on the top of the drop: laser power was 30% (∼120 mW), 10 s of time of exposition. Each sample was analysed in quintuplicates (*n* = 5).

### SERS data analysis

All data processing and visualization were performed within the R software environment (version 4.3.2—“Eye Holes”) for statistical computing and graphics, building on the package hyperSpec.^22^ First, a univariate calibration model using ordinary least squares (OLS) was established by the regression of the integrated areas of the most intense ERG band at 484 cm^−1^ against the known concentration of the target analyte. A further OLS model was then established by replacing the integrated band area of ERG with the band area ratio of ERG to the IS (ERG/5A2MBI). The calibration model was characterized following the requirement of the most recent EURACHEM guidelines on planning and reporting method validation studies^23,24^ on ERG-spiked human serum, which was used as blank matrix. The detection (LOD) and quantitation (LOQ) limits were calculated following the DIN 32645 methodology (equivalent to ISO 11843) using the calibration straight line, as implemented in the chemCal package.^25^

### LC-MS/MS method development and optimisation

The methodology adopted for sample analysis was first optimised based on select standard LC methods in use by the Centre for Metabolomics Research (CMR, University of Liverpool) in conjunction with both low- and high-resolution mass spectrometers. Details of the activities can be found in ESI.†

#### Sample preparation for LC-MS/MS measurements

A random selection of human sera was made from Husermet samples detailed in ref. 18. An initial 5 mM stock solution of ERG was prepared in LC/MS grade water in a 5 mL Eppendorf tube; using a 2.0 mg aliquot from the previously unopened TRC-E600000 sample, weighed on an Ohaus Adventurer balance. An aliquot from this stock was then taken to produce a 0.5 mM solution in 50 : 50 methanol : water, which in turn had an aliquot taken to produce a 0.05 mM (50 μM) solution in 50 : 50 methanol : water. Starting from the 50 μM solution, a series of 1 : 1 dilutions were undertaken to create spike standards of 50 μM, 25 μM, 12.5 μM, *etc.*, down to 0.0977 μM, in addition to a blank “spike” of 0 μM, all in 50 : 50 methanol : water. Including the 0 μM solution, a total of 11 standards of ERG were produced. Due to the need of a relatively large volume of each spike standard for the purposes of this study, all spike standards were prepared in 15 mL centrifuge tubes (Greiner Bio-One CellStar 18826, Greiner Bio-One Ltd UK).

### SERS method validation

Each serum sample to be analysed for the SERS method validation was first removed from a −80 °C freezer, thawed in a refrigerator (∼4 °C) and then vortexed to ensure homogeneity. Aliquots were then taken into two 2 mL Eppendorf tubes: one aliquot (∼1.5 mL) to be used for sample preparation by the University of Liverpool (UoL), the second (∼1 mL) to be provided to the University of Trieste (UoT) for blind analysis. The aliquots were then returned to a −80 °C freezer for storage. This approach allowed for the number of freeze thaw cycles for the serum to be matched in both UoL and UoT.

#### Serum extraction with standard addition spikes

At UoL each serum to be analysed was later rethawed in a refrigerator, vortexed for homogeneity and then had eleven 100 μL aliquots transferred to empty 2 mL Eppendorf safe lock tubes held in a rack on an ice bath. To each of these tubes 300 μL of ultra-cold (∼−40 °C) methanol was added to precipitate proteins thereby facilitating polar metabolite extraction. Next, 100 μL aliquots of ERG standards (11 in total: 0–50 μM) were then added to these tubes. The tubes were then vortexed, to ensure full protein precipitation, mixing and extraction, before being centrifuged for 20 min at 4 °C and 17 000*g* on a Thermo Scientific Fresco Heraeus 17 centrifuge (Thermo Electron LED GmbH, Germany). After centrifugation, 300 μL aliquots of the ‘supernatants’ (*i.e.*, above the now pelletized precipitated proteins) were transferred from each tube to 1.5 mL Eppendorf Safe-Lock tubes and the content subsequently dried to completion using a Scanvac Maxi system (Labogene A/S, Denmark), following which the tubes were returned to a −80 °C freezer for short-term storage before analysis.

#### Sera extract reconstitution

The tubes containing the dried extracts were recovered from the −80 °C freezer. 100 μL of LCMS water was added to each tube, after which they were vortexed to ensure dissolution and mixing of the tube content, then centrifugated for 20 min at 4 °C and 17 000*g*. Finally, aliquots of the content from each tube were transferred to individual glass vials fitted with 300 μL fused inserts (Chromatography Direct VI-04-12-02RVAE), loaded into sample racks and placed into the autosampler (set to 4 °C) of a Thermo Vanquish UHPLC system (Thermo Scientific, DionexSoftron GmbH, Germany) in preparation for analysis.

#### Data acquisition and analysis

Data acquisition for all samples was undertaken on a Thermo Vanquish UHPLC system in conjunction with a Thermo Orbitrap IDX mass spectrometer (ThermoFisher Scientific, U.S.A.). A standard CMR reversed phase UHPLC method was utilised,^26^ which briefly involved a 2 μL sample injection into a 0.4 μL min^−1^ flow gradient of mobile phase A (water + 0.1% formic acid) and mobile phase B (methanol + 0.1% formic acid) in conjunction with a Thermo Hypersil Gold aQ 100 × 2.1 mm, 1.9 μm column set at 50 °C. Mass spectrometry data were acquired in ESI positive ion mode, using a precursor ion targeted (*m*/*z* 230.0959) MS^2^ instrument method and an HCD Collision Energy (%) value of 20. To minimise errors, two sets of sample injections were run for each individual serum, with each set of injections being run as a block (*i.e.*, one serum at a time) in order of spike concentration (starting with the lowest concentration spike of 0 μM and ending with the highest spike at 50 μM). Blank injections were run between the injection sets of serum. The acquired MS-MS data were processed using Thermo Tracefinder 5.1 software, to establish peak areas for the ERG fragment ions of interest (*m*/*z* 186 and 127), with the results subsequently transferred to Microsoft Excel for ease of data review, assessment, plus the calculations of the ERG concentration present in each raw serum.

#### Statistical analysis

The SERS method was validated by comparing the results obtained for the same serum samples by the UHPLC-MS/MS reference method. The agreement between the two methods was investigated using mean bias, limits of agreement (Bland–Altman analysis), paired *t*-test, and Lin's concordance correlation coefficient (CCC) using the SimplyAgree package.^27^

## Results and discussion

Optimization of SERS parameters is typically required to attain the most favourable conditions, ensure reproducibility, and achieve a proper signal enhancement.^28^ The sensitivity of SERS relies on the interaction between analytes and the surface of the SERS substrate, as well as the presence of an aggregation agent. We initially investigated different colloidal substrates commonly used for SERS analysis. Initial analysis using hydroxylamine-reduced silver nanoparticles (hAgNPs),^29^ and citrate-reduced gold nanoparticles (cAuNPs),^30^ yielded unsatisfactory outcomes (data not shown). Therefore, we decided to use only citrated-reduced silver nanoparticles (cAgNPs) as SERS substrate.

### SERS internal standard

**Fig. 3:**
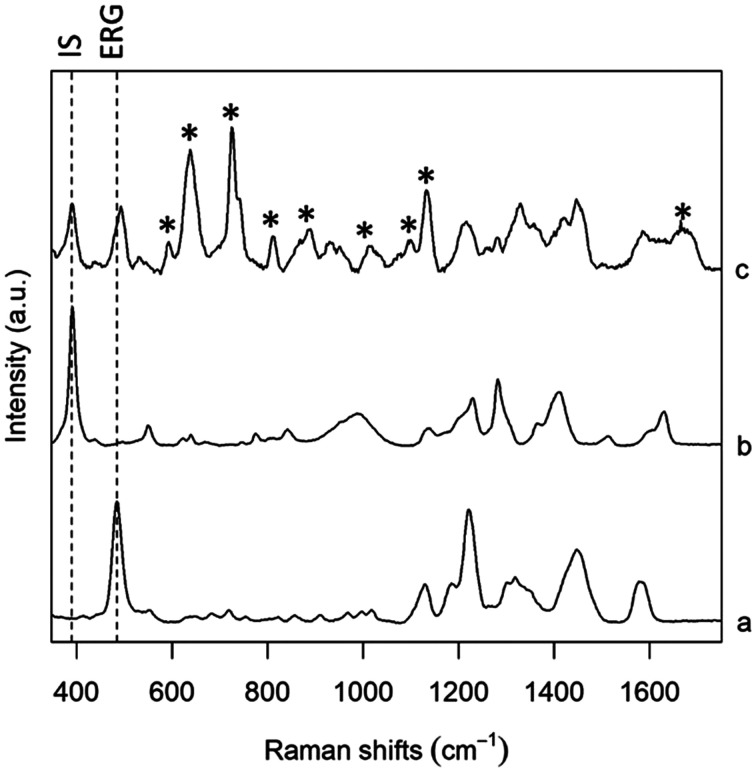
SERS spectra of (a) ERG in PBS, (b) 5A2MBI (IS) in PBS, and (c) serum spiked with both ERG and IS. The intensity of each spectrum was scaled to ease the comparison of spectral features. Bands labelled with * are due to serum components. Dashed lines show IS and ERG bands used for calibration.

A good IS for a SERS method should have an interaction with the substrate as close as possible to that of the analyte of interest, and thus the choice is limited to molecules that are structurally similar to the analyte. Isotopically substituted molecules are excellent IS,^17^ but they can be rather expensive, as they are often specifically produced upon request. Thus, in this study we rather focused on molecules having a structure as close as possible to ERG. In particular, we focused on the molecular moiety that is supposed to interact with metallic SERS substrates. Although a detailed analysis of the ERG-metal adsorption mode and geometry is not available, it is reasonable to assume, based on the well know high binding affinity of thiols for Ag and Au, that the mercaptoimidazole moiety of ERG is the one predominantly involved in bonding the metal substrates.^31^ Three molecules were selected, based on their structural similarity with ERG, as well as from their SERS spectra found in literature, and tested: 2-mercaptoimidazole (2-MI), 2-mercaptobenzimidazole (2-MBI) and 5-amino-2-mercaptobenzimidazole (5A2MBI); all of these can be detected by SERS using the same experimental conditions used for ERG, but they present SERS bands at a slightly different Raman shift than ERG, making their use as IS viable. After an initial phase of screening, where all these analytes were tested in PBS solutions, in absence and then in presence of ERG (data not shown), 5A2MBI was chosen as preferred IS, thanks to its characteristic intense band centred at 391–393 cm^−1^, well differentiated from the main characteristic band from ERG at 484 cm^−1^ (Fig. 3). The similarity between SERS spectra of ERG and those of the IS candidates further confirms our initial assumption about the mercaptoimidazole moiety of ERG as the one being involved in the interaction with the Ag nanoparticles surface used as SERS substrate. The research for the right concentration of IS to use in different protocols has been conducted in solution with PBS and in serum, and after a screening it was decided to use it at 0.25 μM, a concentration that guarantees that the intensity of the IS band was clearly observed, while not interfering with ERG bands for every studied ERG concentration level; normalization with a high intensity value for the IS band would take to underestimate the real measures.

### Quantitative detection of ERG

**Fig. 4:**
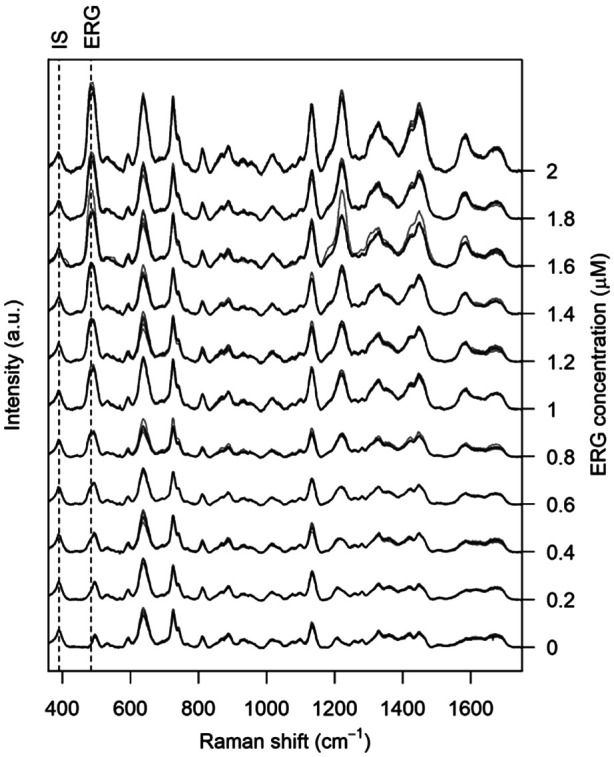
SERS spectra of serum spiked with ERG (at different concentrations) and IS. Dashed lines show IS and ERG bands used for calibration.

**Fig. 5:**
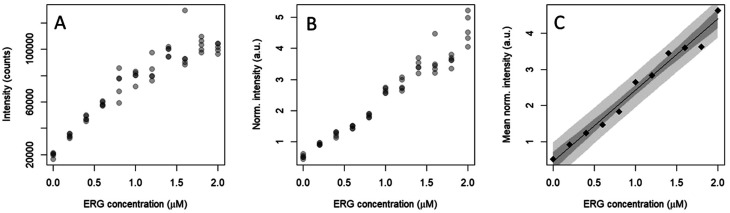
Plots depicting the SERS intensity of spiked human serum samples *vs.* ERG concentration, calculated as area of the ERG band at 485 cm^−1^ (A), as the ratio between the area of the ERG band at 485 cm^−1^ and that of the IS at 390 cm^−1^ (normalized intensity: B), and as normalized intensity mean (C).

Under the optimal detection conditions, the SERS method established in this paper was used to build a regression model to quantify ERG in a physiologically relevant concentration range, in accordance with human metabolome database (HMDB) reported ranges of normal concentrations (https://hmdb.ca/metabolites/HMDB0003045). As shown in Fig. 4, the characteristic band for ERG used in the analysis was at 484 cm^−1^, tentatively assigned, on the basis of available literature, to the to a N–C–S bending mode mixed with a C–S stretching. The improvement of the regression model upon IS normalization is evident (Fig. 5), as it was beneficial to reduce the variability for each concentration level and to constituting a linear trend between normalized intensity and ERG concentration. However, the variance of the normalized intensity values slightly increased with the concentration of calibration standards, suggesting a certain degree of heteroscedasticity. A prerequisite to building a calibration curve using OLS regression is the linear relationship between the concentration and the SERS signal of ERG.

Fig. S1† summarizes the results obtained by applying different linearity tests. As suggested by Raposo,^32^ the deviations calculated from back-calculated concentrations (%RE) can be considered as a key numerical parameter to infer about linearity. Although a polynomial model could fit better, the linear model was selected due to easy interpretation and calculations because the fitness for purposes is achieved. The %REs of back-calculated deviations in the working range of the calibration were all within the proposed acceptance limits (<20%). The limit of detection (LOD) was 0.41 μM, and the limit of quantification (LOQ) was found to be 0.71 μM (Table 1). This value was acceptable regarding the defined physiologically relevant concentration range.

**Table 1:** Figures of Merit (FoM) of the SERS method for the determination of ergothioneine

FoM	
LOD	0.41 μM
LOQ	0.71 μM
Working range	0.71–2 μM

### Blind test measurements of ERG

**Fig. 6:**
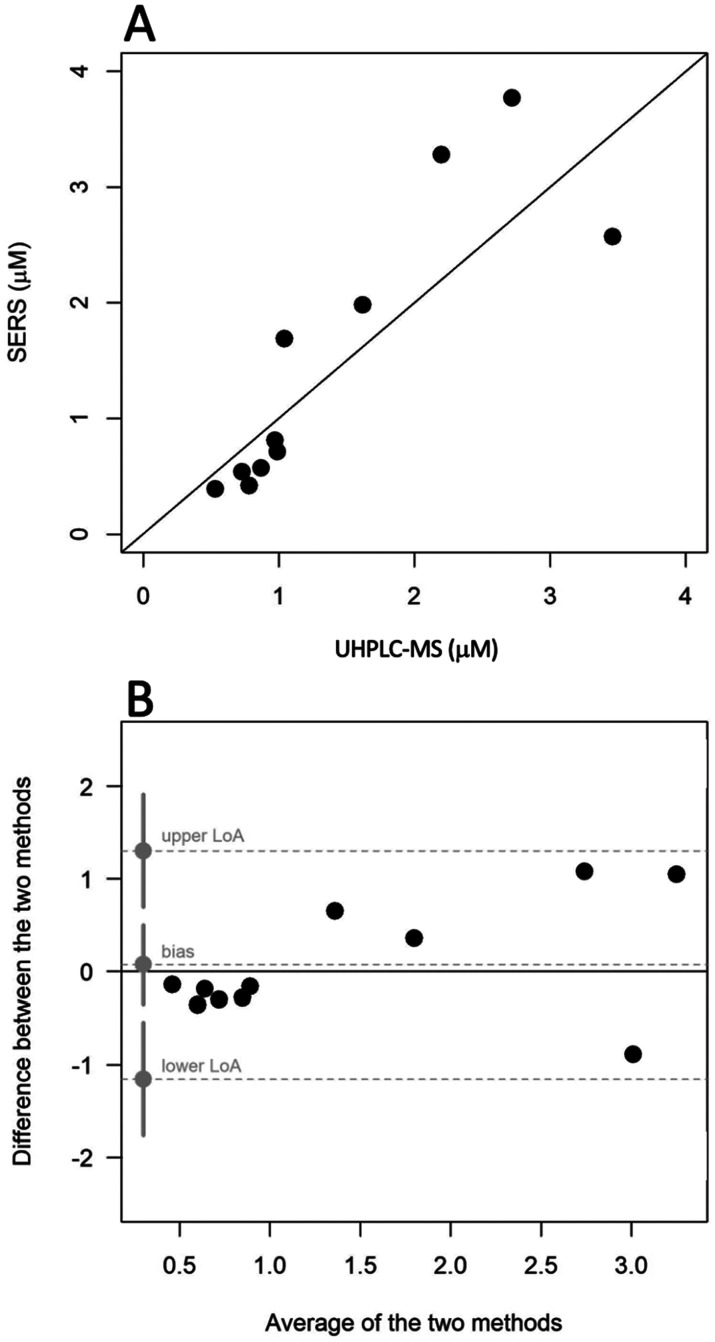
Agreement between SERS and UHPLC-MS measurements of ERG in real serum samples. (A) scatter plot of SERS *vs.* UHPLC-MS; (B) Bland–Altman plot reporting bias, upper and lower limits of agreement (LoA), together with their confidence intervals (95% for bias, 90% for upper and lower LoA).

Due to the lack of certified reference materials for ERG in serum, a blind test experiment on 11 real serum samples was performed to validate the IS-SERS method using UHPLC-MS as the primary reference method. The levels of ERG in these test samples were known to UoL after LC-MS, but these levels were not revealed to UoT until after the SERS analyses. Fig. 6A depicts the relation between the ERG values obtained by the two methods. The concordance correlation coefficient (CCC), measures closeness of the point in the scatterplot to the identity line (*y* = *x*) between the results obtained by SERS and UHPLC-MS analysis. The relatively high value of the CCC (0.83) provided a first indication of the congruence of the methods for the detection of ERG in human serum. The CCC is considered as a comprehensive figure of merit for a model that evaluates trueness and precision at the same time, as in this case, where the real trueness could not be directly assessed. Nevertheless, the rather broad 95% confidence interval (0.55–0.94) made it difficult to conclude about between-method differences. Paired *t*-test analysis also revealed no statistically significant difference in the mean ERG measured by both methods (*p* = 0.919). Bland–Altman analysis for agreement (Fig. 6B) found a mean absolute bias of 1% (0.02 μM; 95% limits of agreement, LoA, ranging from −1.22 μM to 1.26 μM), considerably lower than acceptable limits of 20%.^33^ The line of equality fell inside in the 95% confidence interval for the bias (−0.41–0.44 μM) allowing us to safely claim that, on average, the differences in ERG measurements were negligible and random across the range.

Despite the limited number of samples, these findings indicate a satisfactory level of agreement between the two methods, showcasing the effectiveness of the IS-SERS methodology on real samples. Given the preliminary nature of this study, we can infer that the proposed IS-SERS method has the potential to become a clinically valuable and reliable analytical tool: the extent to which two measurements can differ before causing difficulties is not a matter of statistics, but rather requires clinical judgement on a case-by-case basis.

## Conclusions

In this work we have described a new spectroscopic method involving surface enhanced Raman scattering (SERS) for the determination of the dietary amino acid ergothioneine (ERG) in human serum. In order to do this, we have developed a novel direct method based on a highly specific internal standard (5-amino-2-mercaptobenzimidazole, 5A2MBI), with low likelihood for overlap with the ERG fingerprint spectral bands. This method effectively corrects for SERS intensity variations caused by unavoidable differences in the number of nanoparticles in the Raman collection voxel, along with any differences in laser power, as well as the complex composition and dynamic of the serum samples. Moreover, the proposed method also showed good quantitative accuracy for SERS analysis of real samples which were analysed blind. In this process high confidence in these results can be made as the levels of ERG in these test samples were known to UoL after LC-MS, and these levels were not revealed to UoT until after the SERS analyses.

## Ethical statement

Our study used stored biological specimens (human sera) obtained during previously authorised research. The original study, detailed in ref. 18, conformed to the principles set out in the WMA Declaration of Helsinki and the NIH Belmont report and was approved by the Stockport Local Research Ethics Committee.

## Author contributions

S. F. conceptualization, methodology, formal analysis, investigation, writing – original draft, writing – review & editing, data curation. N. G. investigation, writing – review & editing. G. V. investigation. M. W. M. investigation. I. R. investigation. V. S. resources. R. G. resources, writing – review & editing, funding acquisition. A. B. conceptualization, methodology, resources, visualization, writing – original draft, writing – review & editing, supervision, project administration.

## Data availability

Data for this article are available at Zenodo at https://doi.org/10.5281/zenodo.13785348.

## Conflicts of interest

There are no conflicts to declare.

## Supplementary Material

AN-150-D4AN01323A-s001
