# Supplementary material for: Detection and quantification of ergothioneine in human serum using surface enhanced Raman scattering (SERS)
Source: Analyst. 2024 Dec 30;150(3):559–66. doi: 10.1039/d4an01323a (PMC11731495; doi:10.1039/d4an01323a)
Supplement: AN-150-D4AN01323A-s001 [file AN-150-D4AN01323A-s001.pdf]

Supplementary Information (SI) for Analyst.
This journal is © The Royal Society of Chemistry 2024

Detection and quantification of ergothioneine in human serum using surface enhanced Raman scattering (SERS)

Stefano Fornasaro,*[a] Nigel Gotts,[b] Gioia Venturotti,[c] Marina Wright Muelas,[b] Ivayla Roberts,[b] Valter Sergo,[c] Royston Goodacre,[b] and Alois Bonifacio[c]

[a] Department of Chemical and Pharmaceutical Sciences, University of Trieste, via L. Giorgieri 1, 34127 Trieste, Italy.

[b] Centre for Metabolomics Research, Department of Biochemistry, Cell and Systems Biology, Institute of Systems, Molecular, and Integrative Biology, University of Liverpool, Liverpool L69 7ZB, UK.

[c] Department of Engineering and Architecture, University of Trieste, via A. Valerio 6 34127 Trieste, Italy.

Supplementary materials

LCMS Method development and optimisation

Sample preparation

Pure ergothioneine standards for initial MS detection and optimisation

A range of concentration standards, up to 25000 ng/mL (~ 109 µM), of ergothioneine in acetonitrile (ACN) were prepared for subsequent HILIC LCMS analyses via the following process. A small quantity (mg) of Ergothioneine would be transferred into to a 5 mL Eppendorf screw top tube, then dissolved directly in ACN with the assistance of a vortex mixer. The concentration standards were then created via sequential dilution series (starting from this high stock solution) into ACN, into a set of 2 mL Eppendorf safe-lock tubes.

Ergothioneine spikes in "test" serum

Extraction

A 5 mM stock solution of ergothioneine was prepared by dissolving a few milligrams (typically ~5 mg) into the requisite volume (typically 4-5 mL) of water. This solution was then sequentially diluted (into 2 mL tubes) to produce a set of concentration standards in 50:50 methanol:water extending from 100 µM down to 0.1 µM.

Test serum samples were then retrieved from a -80˚C freezer, allowed to thaw in a refrigerator (~4˚C) and subsequently vortexed to ensure homogeneity. 100 µL aliquots of the homogenised serum was then added to a set of empty 2 mL Eppendorf tubes held in a rack on an ice bath. 300 µL aliquots of ultra cold (approx. -40˚C) MeOH were next added to each serum containing tube to facilitate protein precipitation and polar content extraction, after which 100 µL aliquots of one ergothioneine standard solution (from 100µM down to 0µM - blank) was added to one of the individual tubes. The tubes were next vortexed to ensure full protein precipitation and mixing, then centrifuged for 20 min at 4˚C and 17000 *g* on a Thermo Fresco Heraeus 17 centrifuge (to pellet the precipitated content from the extract supernatant).

Next, 300 µL aliquots of the extracted supernatant were transferred from each of the tube into a set of 1.5 mL Eppendorf tubes and their content subsequently dried under vacuum using a Scanvac vacuum centrifuge system. The tubes of dried extract were finally moved to a -80˚C freezer for short term storage.

Reconstitution

The tubes of dried extract were recovered from the -80˚C freezer. 100 µL of water was then added to each tube, following which the tubes were subjected to vortex content mixing (to facilitate dissolution of the dried material) and then centrifugation (to ensure any undissolved material in the tubes would be separated from the reconstituted supernatant). Finally, aliquots of the reconstituted supernatant were transferred to 300 µL glass insert vials for analysis.

LC-MS-MS method development and optimisation

Pure ergothioneine standards

Initial – Thermo TSQ Fortis (Triple Quad)

Positive and negative ion fragmentation transitions for the ergothioneine concentration standards were identified using a Thermo TSQ Fortis mass spectrometer (ThermoFisher Scientific, U.S.A.) in conjunction with a Thermo Vanquish UHPLC system utilising a HILIC solvent gradient (mobile phase A: 95% ACN:water, 10 mM ammonium formate + 0.1% formic acid; mobile phase B: 50% ACN:water, 10 mM ammonium formate + 0.1% formic acid) and a Thermo Accucore 150 Amide-HILIC 100x2.1 mm, 2.6 µm column.

Fragment ion transitions from the parent ion that exhibited good linearity and apparently minimal background interference were established and preferred transitions subsequently selected corresponding to the positive ion mode fragment ions of *m/z* 186 and 127; plus an additional fragment ion with *m/z* 69 as an alternate.

The LoD observed for the ergothioneine fragments in the experiment was around 0.2 µM.

Continued – Thermo Orbitrap IDX

While triple quad instruments typically provide the best dynamic range linearity for quantitation analyses as well as lowest limits of quantitation (LOQs), we have observed that our Orbitrap systems can on a case-by-case basis match or better our in-house triple quad determined LOQs plus exhibit good linearity for fragment ions over low concentration ranges.

To optimise the LCMS/MS analysis methods further we therefore ran tests using a Thermo Orbitrap IDX in conjunction with a Vanquish UHPLC system. The investigation was also extended to cover both HILIC (as previously described) and reversed phase chromatographic approaches; the reversed phase approach being a gradient of mobile phase A (water + 0.1% formic acid) and mobile phase B (methanol + 0.1% formic acid) in conjunction with a Thermo Hypersil Gold aQ 100x2.1 mm, 1.9 µm column.

While MS^2 analyses on the Fortis allows for individually optimized collision parameters for each fragment ion being detected within an MRM (multiple-reaction-monitoring) MS method, such an approach is not available on the IDX. Therefore an investigatory instrument method was first utilised on our IDX to determine the optimum collision energy for detection of fragment ions from the target ergothioneine ion at *m/z* 230.0959 (positive ion mode); incorporating slow injection of a large sample volume into a low mobile phase flow (no column) with rapid stepped test settings of HCD Collision Energy (%) and CID Collision Energy (%) values; HCD (higher-energy collisional dissociation) being a CID (collision-induced dissociation) technique that is associated with Thermo Scientific Orbitrap instruments, whereby

fragmentation is induced in the ion-routing multipole of the instrument as opposed to the ion trap. This was performed for both the HILIC and reversed phase LC methods. The results from this test indicated that for both HILIC and reversed phase, a HCD Collision Energy (%) value of 20 resulted in fragmentation spectra exhibiting optimal expression across all three fragment ions of interest.

Once the optimal HCD Collision Energy value was established, this was then used in a precursor ion targeted MS^2 instrument method to analyse the set of pure ergothioneine concentration standards in order to discern IDX performance; with peak areas of the fragments in the acquired data being determined using Thermo's TraceFinder program. Results suggested that the reversed phase approach (exhibiting albeit weakly retained ergothioneine at ~ 0.71 min retention time (RT)) provided better results and detection limits (seemingly down to less than 0.03 µM) than the HILIC approach (RT ~ 7.1 min).

Given the apparent favourability of the IDX based analysis over that of the Fortis based analysis, supplemented by the IDX's high resolution capability to "separate" potential interferents that may be present in complex samples, the preparatory work using the IDX was extended to the analysis of spiked sera.

Ergothioneine spiked in "test" serum – Thermo Orbitrap IDX

The reconstituted spiked serum extract samples were run on a Thermo Orbitrap IDX mass spectrometer in conjunction with a Vanquish UHPLC system using a reversed phase chromatographic gradient method and precursor ion targeted MS^2 mass spectrometer methods as previously described; the acquired data being subsequently processed via Thermo's TraceFinder program to determine fragment peak areas for the *m/z* 186 and 127 ions (plus also the *m/z* 60 ion).

A matching experiment was also conducted using a HILIC chromatographic method (as previously described) for completeness.

These results confirmed that for the spiked serum extract standards within the IDX suited concentration range, the reverse phase approach produced the better peak shapes and linearity.

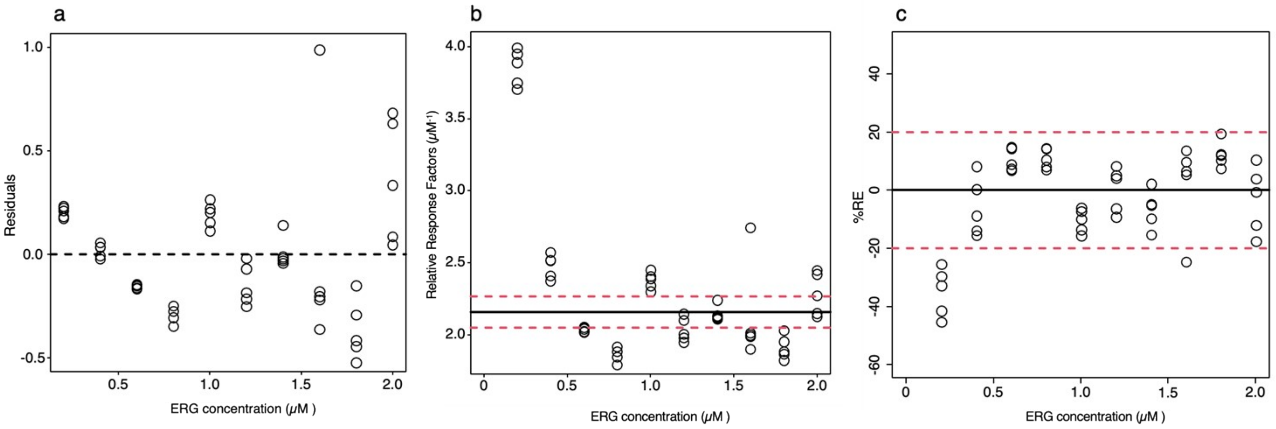

Figure S1. Linearity assessment. a) Residuals plot. b) Sensitivity (response factors) profile. Tolerance limits of +- 5% are reported as red dashed lines c) Relative error (%RE) plot. +- 20% deviations limits are reported as red dashed lines.
